# Synthesis and Biological Evaluations of Betulinic Acid Derivatives With Inhibitory Activity on Hyaluronidase and Anti-Inflammatory Effects Against Hyaluronic Acid Fragment Induced Inflammation

**DOI:** 10.3389/fchem.2022.892554

**Published:** 2022-05-04

**Authors:** Zhujun Luo, Hao He, Tiantian Tang, Jun Zhou, Huifang Li, Navindra P. Seeram, Dongli Li, Kun Zhang, Hang Ma, Panpan Wu

**Affiliations:** ^1^ School of Biotechnology and Health Sciences, Wuyi University, Jiangmen, China; ^2^ International Healthcare Innovation Institute, Jiangmen, China; ^3^ Bioactive Botanical Research Laboratory, Department of Biomedical and Pharmaceutical Sciences, College of Pharmacy, University of Rhode Island, Kingston, NY, United States; ^4^ School of Chemical Engineering, Shanxi Institute of Science and Technology, Jincheng, China; ^5^ Laboratory of Nutrition and Development, Key Laboratory of Major Diseases in Children, Ministry of Education, Beijing, China; ^6^ Beijing Pediatric Research Institute, Beijing Children’s Hospital, Capital Medical University, Beijing, China; ^7^ National Center for Children’s Health, Beijing, China

**Keywords:** betulinic acid, α,β-unsaturated ketene, derivative, hyaluronidase, anti-inflammatory

## Abstract

We previously reported that the structural modifications of pentacyclic triterpenoids including oleanolic acid resulted in enhanced hyaluronidase inhibitory activity but whether this applies to other pentacyclic triterpenoids such as betulinic acid (BA) is unknown. Herein, we synthesized BA derivatives with an α,β-unsaturated ketene moiety and evaluated for their: 1) hyaluronidase inhibitory activity and, 2) anti-inflammatory effects against lipopolysaccharides (LPS) induced inflammation. Compared to BA, the BA derivatives exerted improved anti-hyaluronidase activity (26.3%–72.8% vs. 22.6%) and anti-inflammatory effects by reducing nitrite production in BV2 cells (3.9%–46.8% vs. 3.4%) and RAW264.7 cells (22.7%–49.2% vs. 20.4%). BA derivatives inhibited LPS-induced production of pro-inflammatory cytokines in THP-1 cells (15.2%–22.4%). BA derivatives also exerted promising anti-inflammatory effects against hyaluronic acid fragment induced nitrite production (8.6%–35.6%) in THP-1 cells. BA derivatives showed augmented anti-hyaluronidase and anti-inflammatory effects but further biological evaluations using *in vivo* models are warranted to confirm their efficacy.

## Graphical Abstract

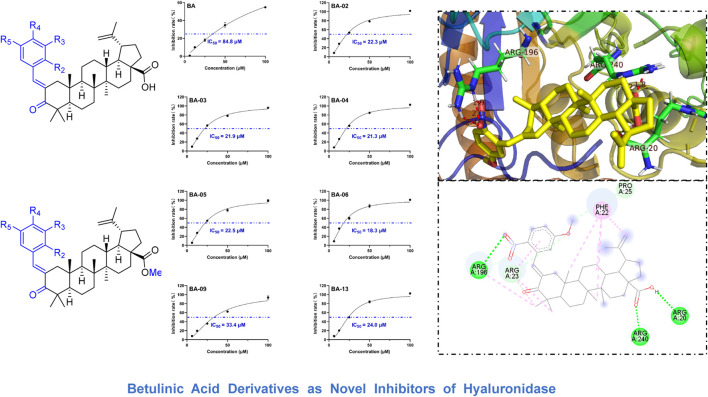

## Highlights

• Novel betulinic acid (BA) derivatives with α,β-unsaturated ketene moiety
• BA derivatives exhibit augmented anti-hyaluronidase activity
• BA derivatives exert anti-inflammatory effects in murine macrophage and human monocyte cells
• BA derivatives inhibit hyaluronic acid fragment induced inflammation in human monocytes

## Introduction

Hyaluronic acid (HA) is a naturally occurring polysaccharide produced in the cell plasma membrane and distributed in all living organisms (Fallacara et al., 2018). HA has a linear structure with numerous repeating units consisting of anionic, nonsulfated disaccharides known as glycosaminoglycan (GAG). Each GAG disaccharide unit contains an amino sugar (N-acetyl-galactosamine or N-acetyl-glucosamine) and a uronic sugar (glucuronic acid, iduronic acid, or galactose) linked by β-1,3-glycosidic bonds (Girish and Kemparaju, 2007). The GAG two-sugar repeating units are connected by 1,3-glycosidic bonds to form a varying length of HA, which can reach to a high molecular weight (HMW) form with up to 2,50,000 GAGs (Girish and Kemparaju, 2007). HA is negatively charged in an aqueous solution, which enables it to attract water molecules and display a non-Newtonian, shear-thinning, and viscoelastic rheological profile. Due to its unique structural characteristics, HA has several critical biological functions, such as maintaining the elastoviscosity of extracellular matrix (ECM) in connective tissues and lubricating movable parts of the body (Price et al., 2007). In addition, HA plays a pivotal regulatory role in various cellular functions (e.g., cell growth, mitosis, and migration) and physiological conditions including tumor development and inflammation (Fallacara et al., 2018). Notably, HA’s structural property, especially its size, and the synthesis-degradation rate, is a key factor for its modulatory effects in the development of inflammation (Jiang et al., 2011). Reported studies suggest that HA can exert both pro- and anti-inflammatory effects and this opposite effect is mediated by its length. Intact high molecular weight HA (HMWHA; >1 × 10^6^ Da) exerts anti-inflammatory effects by suppressing the recruitment of inflammatory cells and reducing the levels of pro-inflammatory cytokines (Quero et al., 2013; Litwiniuk et al., 2016). On the contrary, HMWHA can be degraded into smaller fragments known as low molecular weight HAs (LMWHAs; 2 × 10^4^–1 × 10^6^ Da), which stimulates the production of pro-inflammatory cytokines and chemokines (Litwiniuk et al., 2016; Fallacara et al., 2018) The degradation of HMWHA can be mediated by two types of mechanisms: 1) non-specific oxidative damage by reactive oxygen species (e.g., free radicals) generated during tissue injury and infection; and 2) fragmentation catalyzed by a group of enzymes named hyaluronidases (Fallacara et al., 2018). Hyaluronidase (HAase) is the key enzyme catalyzing the cleavage of the β-1,4-glycosidic bonds of HA to produce small HA fragments (<2 × 10^4^ Da). HA fragments can bind to a set HA cell surface receptor, such as CD44 and toll-like receptors, and subsequently triggers signaling cascades to exacerbate inflammation (Quero et al., 2013). Thus, HAase inhibitors which have the potential to suppress the generation of HA fragments are regarded as promising anti-inflammatory agents (Girish et al., 2009).

Phytochemicals including triterpenes from medicinal plants show great promise as HAase inhibitors. For instance, a group of pentacyclic triterpenoids, including a representative compound, namely, ursolic acid (UA), from *Prismatomeris tetrandra* showed inhibitory effects on the activity of HAase. Furthermore, chemical modifications (i.e., acetylation and methylation) of the UA skeleton resulted in the generation of new UA analogues with enhanced anti-HAase activity (Abdullah et al., 2016). In addition, an extract of *Carissa carandas* leaf containing a high level of UA was reported to inhibit the activity of HAase and reduce the level of inflammatory biomarkers, such as nuclear factor kappa B, in human monocytes (Neimkhum et al., 2021). Given these previously published data, our laboratory initiated a project to screen for small molecule based HAase inhibitors from natural resources and a triterpenoid, namely, oleanolic acid (OA), as a “lead compound”. We showed that chemical synthesis of OA derivatives with indole moieties led to HAase inhibitors with augmented activity (He et al., 2021). Betulinic acid (BA), another subtype of pentacyclic triterpenoid, has been reported to show anti-HAase and anti-inflammatory effects (Tsai et al., 2011; Abdullah et al., 2016; Ou et al., 2019). However, it is not clear whether the inhibitory effect of BA on HAase can be improved by chemical modifications, nor whether BA based HAase inhibitors can inhibit HA fragment induced inflammation. Herein, we initiated this study to 1) synthesize novel BA derivatives and assess their anti-HAase activity using *in vitro* and in silico methods; and 2) evaluate the anti-inflammatory effects of BA derived HAase inhibitors against HA-fragment induced inflammation using a panel of murine and human cells.

## General Procedures for the Synthesis of BA Derivatives

### Preparation of BA Derivatives BA-O and BA-O-Me

BA was dissolved in acetone at 0°C; Jones reagent was added to the reaction mixture drop-wise until the solution color was a stable light brown color, which implied that the Jones reagent was in slight excess to oxidize the C-3 hydroxyl group into a ketone to produce the intermediate BA-O. Then the BA-O was dissolved in DMF. K_2_CO_3_ and CH_3_I were added and the mixture was stirred overnight at room temperature to obtain the BA derivative BA-O-Me.

### General Procedure for the Preparation of BA Derivatives (BA-01 to BA-22)

Derivatives BA-01∼BA-13 could be prepared by Claisen Schmidt condensation of intermediate BA-O with corresponding aldehydes in the presence of ethanolic potassium hydroxide in good yield at room temperature. The same condition of reactions starting with BA-O-Me led to derivatives BA-14∼BA-22. All the results were detailed below.

### Synthesis of BA Derivative BA-01–BA-13

**BA-01** *(1R,3aS,5aR,5bR,7aR,11aR,11bR,13aR,13bR)-10-((E)-3-fluorobenzylidene)-5a,5b,8,8,11a-pentamethyl-9-oxo-1-(prop-1-en-2-yl)icosahydro-3aH-cyclopenta[a]chrysene-3a-carboxylic acid* (BA-01, C_37_H_49_FO_3_). According to the general procedure, derivative BA-01 was prepared by Claisen Schmidt condensation of intermediate BA-O with 3-fluorobenzaldehyde in the presence of ethanolic potassium hydroxide at room temperature. The residue was purified by flash chromatography (eluent: petroleum ether: ethyl acetate = 10: 1) to afford BA-01 as a white solid with a yield of 90%. ^1^H NMR (600 MHz, Chloroform-*d*) *δ* 7.32 (dd, *J* = 3.0, 1.5 Hz, 1H), 7.27 (td, *J* = 8.0, 6.0 Hz, 1H), 7.08 (d, *J* = 7.8 Hz, 1H), 7.00 (dt, *J* = 10.2, 2.1 Hz, 1H), 6.93 (td, *J* = 8.4, 2.6 Hz, 1H), 4.79–4.48 (m, 2H), 3.01–2.85 (m, 2H), 2.22–2.11 (m, 2H), 2.11–2.06 (m, 1H), 1.95–1.86 (m, 2H), 1.60 (d, *J* = 34.7 Hz, 5H), 1.47–1.29 (m, 11H), 1.24–1.11 (m, 3H), 1.04 (s, 3H), 1.02 (s, 3H), 0.93 (s, 3H), 0.87 (s, 3H), 0.69 (s, 3H). ^13^C NMR (151 MHz, CDCl_3_) *δ* 208.09, 181.99, 161.78, 150.38, 138.08, 135.92, 135.39, 129.92, 126.07, 116.57, 115.23, 109.79, 56.43, 52.82, 49.15, 48.35, 46.85, 45.24, 44.31, 42.51, 40.52, 38.43, 37.02, 36.54, 33.01, 32.05, 30.59, 29.67, 29.42, 25.54, 22.33, 21.63, 20.33, 19.47, 15.81, 15.47, 14.61. HRMS (ESI): C_37_H_49_FNaO_3_ (583.3558) [M + Na]^+^ = 583.3558.

**BA-02** *(1R,3aS,5aR,5bR,7aR,11aR,11bR,13aR,13bR)-10-((E)-2-bromobenzylidene)-5a,5b,8,8,11a-pentamethyl-9-oxo-1-(prop-1-en-2-yl)icosahydro-3aH-cyclopenta[a]chrysene-3a-carboxylic acid* (BA-02, C_37_H_49_BrO_3_). According to the general procedure, derivative BA-02 was prepared by Claisen Schmidt condensation of intermediate BA-O with 2-bromobenzaldehyde in the presence of ethanolic potassium hydroxide at room temperature. The residue was purified by flash chromatography (eluent: petroleum ether: ethyl acetate = 10: 1) to afford BA-02 as a white solid with a yield of 71%. ^1^H NMR (600 MHz, Chloroform-*d*) *δ* 7.61 (dd, *J* = 8.0, 1.2 Hz, 1H), 7.50–7.47 (m, 1H), 7.33 (td, *J* = 7.5, 1.2 Hz, 1H), 7.22 (dd, *J* = 7.8, 1.6 Hz, 1H), 7.18 (td, *J* = 7.7, 1.7 Hz, 1H), 4.66 (dt, *J* = 71.4, 1.6 Hz, 2H), 2.99 (td, *J* = 10.8, 4.9 Hz, 1H), 2.81 (dd, *J* = 16.0, 1.5 Hz, 1H), 2.28 (dt, *J* = 13.0, 3.4 Hz, 1H), 2.21 (td, *J* = 12.3, 3.6 Hz, 1H), 2.02–1.94 (m, 3H), 1.66 (d, *J* = 32.6 Hz, 5H), 1.46 (tdd, *J* = 22.8, 16.7, 13.3 Hz, 10H), 1.32 (d, *J* = 10.6 Hz, 1H), 1.26–1.21 (m, 2H), 1.17 (s, 3H), 1.13 (s, 3H), 1.09–1.01 (m, 1H), 0.99 (s, 3H), 0.95 (s, 3H), 0.80 (s, 3H). ^13^C NMR (151 MHz, CDCl_3_) *δ* 208.06, 181.59, 150.48, 136.57, 136.40, 135.99, 132.86, 130.12, 129.42, 127.04, 124.81, 109.65, 56.38, 53.29, 49.13, 48.36, 46.81, 45.71, 43.36, 42.50, 40.54, 38.41, 37.00, 36.85, 33.17, 32.04, 30.56, 29.65, 28.91, 25.49, 22.42, 21.46, 20.19, 19.43, 15.73, 15.54, 14.60. HRMS (ESI): C_37_H_49_^79^BrNaO_3_ (643.2757) [M + Na]^+^ = 643.2757, C_37_H_49_^81^BrNaO_4_ (645.2737) [M + Na]^+^ = 645.2737.

**BA-03** *(1R,3aS,5aR,5bR,7aR,11aR,11bR,13aR,13bR)-10-((E)-5-methoxy-2-nitrobenzylidene)-5a,5b,8,8,11a-pentamethyl-9-oxo-1-(prop-1-en-2-yl)icosahydro-3aH-cyclopenta[a]chrysene-3a-carboxylic acid* (BA-03, C_38_H_51_NO_6_). According to the general procedure, derivative BA-03 was prepared by Claisen Schmidt condensation of intermediate BA-O with 5-methoxy-2-nitrobenzaldehyde in the presence of ethanolic potassium hydroxide at room temperature. The residue was purified by flash chromatography (eluent: petroleum ether: ethyl acetate = 10: 1) to afford BA-03 as a white solid with a yield of 79%. ^1^H NMR (600 MHz, Chloroform-*d*) *δ* 8.20 (d, *J* = 9.1 Hz, 1H), 7.60 (d, *J* = 2.6 Hz, 1H), 6.93 (dd, *J* = 9.2, 2.8 Hz, 1H), 6.67 (d, *J* = 2.8 Hz, 1H), 4.77–4.50 (m, 2H), 3.91 (s, 3H), 2.97 (td, *J* = 10.8, 5.0 Hz, 1H), 2.63 (dd, *J* = 15.8, 1.4 Hz, 1H), 2.27 (dt, *J* = 12.9, 3.3 Hz, 1H), 2.20 (td, *J* = 12.3, 3.6 Hz, 1H), 1.97 (qd, *J* = 9.1, 8.0, 3.0 Hz, 2H), 1.87–1.78 (m, 1H), 1.69–1.58 (m, 5H), 1.52–1.44 (m, 3H), 1.43–1.35 (m, 5H), 1.33 (d, *J* = 7.0 Hz, 1H), 1.29–1.24 (m, 2H), 1.22 (dt, *J* = 13.2, 3.2 Hz, 2H), 1.18 (s, 3H), 1.13 (s, 3H), 1.03–0.98 (m, 1H), 0.96 (s, 3H), 0.93 (s, 3H), 0.80 (s, 3H). ^13^C NMR (151 MHz, CDCl_3_) *δ* 207.90, 181.11, 163.16, 150.59, 140.90, 135.58, 135.29, 134.92, 127.63, 115.75, 113.33, 109.58, 56.35, 56.07, 53.41, 49.07, 48.25, 46.81, 45.97, 43.04, 42.50, 40.55, 38.36, 36.98, 33.18, 32.02, 30.55, 29.71, 29.63, 28.64, 25.46, 22.41, 21.41, 20.12, 19.43, 15.73, 15.55, 14.57. HRMS (ESI): C_38_H_51_NNaO_6_ (640.3609) [M + Na]^+^ = 640.3609.

**BA-04** *(1R,3aS,5aR,5bR,7aR,11aR,11bR,13aR,13bR)-10-((E)-5-fluoro-2-nitrobenzylidene)-5a,5b,8,8,11a-pentamethyl-9-oxo-1-(prop-1-en-2-yl)icosahydro-3aH-cyclopenta[a]chrysene-3a-carboxylic acid* (BA-04, C_37_H_48_FNO_5_). According to the general procedure, derivative BA-04 was prepared by Claisen Schmidt condensation of intermediate BA-O with 5-fluoro-2-nitrobenzaldehyde in the presence of ethanolic potassium hydroxide at room temperature. The residue was purified by flash chromatography (eluent: petroleum ether: ethyl acetate = 10: 1) to afford BA-04 as a white solid with a yield of 88%. ^1^H NMR (600 MHz, Chloroform-*d*) *δ* 8.20 (dd, *J* = 9.1, 5.0 Hz, 1H), 7.54 (d, *J* = 2.8 Hz, 1H), 7.17 (ddd, *J* = 9.5, 7.1, 2.8 Hz, 1H), 6.96 (dd, *J* = 8.5, 2.8 Hz, 1H), 4.74–4.55 (m, 2H), 2.97 (td, *J* = 10.7, 5.0 Hz, 1H), 2.66–2.52 (m, 1H), 2.27 (dt, *J* = 13.0, 3.4 Hz, 1H), 2.20 (td, *J* = 12.3, 3.6 Hz, 1H), 2.01–1.94 (m, 2H), 1.88 (dd, *J* = 15.9, 3.0 Hz, 1H), 1.64 (d, *J* = 34.2 Hz, 5H), 1.55–1.32 (m, 11H), 1.30–1.18 (m, 3H), 1.18 (s, 3H), 1.13 (s, 3H), 1.04–0.97 (m, 1H), 0.97 (s, 3H), 0.94 (s, 3H), 0.80 (s, 3H). ^13^C NMR (151 MHz, CDCl_3_) *δ* 207.43, 181.64, 163.71, 150.42, 144.10, 136.70, 135.63, 132.95, 127.83, 117.69, 115.82, 109.70, 56.36, 53.36, 49.07, 48.24, 46.82, 45.95, 43.10, 42.50, 40.55, 38.36, 37.00, 36.98, 33.13, 32.02, 30.53, 29.62, 28.67, 25.39, 22.41, 21.42, 20.11, 19.39, 15.70, 15.54, 14.56. HRMS (ESI): C_37_H_48_FNNaO_5_ (628.3409) [M + Na]^+^ = 628.3408.

**BA-05** *(1R,3aS,5aR,5bR,7aR,11aR,11bR,13aR,13bR)-10-((E)-2-methoxybenzylidene)-5a,5b,8,8,11a-pentamethyl-9-oxo-1-(prop-1-en-2-yl)icosahydro-3aH-cyclopenta[a]chrysene-3a-carboxylic acid* (BA-05, C_38_H_52_O_4_). According to the general procedure, derivative BA-05 was prepared by Claisen Schmidt condensation of intermediate BA-O with 2-methoxybenzaldehyde in the presence of ethanolic potassium hydroxide at room temperature. The residue was purified by flash chromatography (eluent: petroleum ether: ethyl acetate = 10: 1) to afford BA-05 as a white solid with a yield of 74%. ^1^H NMR (600 MHz, Chloroform-*d*) *δ* 7.78–7.74 (m, 1H), 7.30 (ddd, *J* = 19.2, 7.7, 1.7 Hz, 2H), 6.98 (t, *J* = 7.5 Hz, 1H), 6.90 (d, *J* = 8.3 Hz, 1H), 4.68 (d, *J* = 68.0 Hz, 2H), 3.83 (s, 3H), 3.00 (ddd, *J* = 15.9, 5.7, 3.1 Hz, 2H), 2.33–2.26 (m, 1H), 2.23 (td, *J* = 12.3, 3.6 Hz, 1H), 2.11 (dd, *J* = 16.1, 3.1 Hz, 1H), 2.05–1.93 (m, 2H), 1.68 (d, *J* = 33.8 Hz, 5H), 1.56–1.47 (m, 2H), 1.49–1.39 (m, 8H), 1.25 (ddd, *J* = 16.3, 10.4, 4.1 Hz, 3H), 1.14 (s, 3H), 1.13 (s, 3H), 1.08 (dd, *J* = 13.1, 4.3 Hz, 1H), 1.01 (s, 3H), 0.96 (s, 3H), 0.79 (s, 3H). ^13^C NMR (151 MHz, CDCl_3_) *δ* 207.89, 182.06, 158.39, 150.50, 133.72, 133.24, 129.88, 129.83, 125.01, 120.02, 110.69, 109.66, 56.43, 55.49, 53.10, 49.16, 48.40, 46.83, 45.30, 43.99, 42.50, 40.53, 38.45, 37.02, 36.64, 33.17, 32.07, 30.59, 29.67, 29.29, 25.57, 22.53, 21.52, 20.29, 19.46, 15.71, 15.53, 14.61. HRMS (ESI): C_38_H_52_NaO_4_ (595.3758) [M + Na]^+^ = 595.3758.

**BA-06** *(1R,3aS,5aR,5bR,7aR,11aR,11bR,13aR,13bR)-10-((E)-4-fluoro-2-nitrobenzylidene)-5a,5b,8,8,11a-pentamethyl-9-oxo-1-(prop-1-en-2-yl)icosahydro-3aH-cyclopenta[a]chrysene-3a-carboxylic acid* (BA-06, C_37_H_48_FNO_5_). According to the general procedure, derivative BA-06 was prepared by Claisen Schmidt condensation of intermediate BA-O with 4-fluoro-2-nitrobenzaldehyde in the presence of ethanolic potassium hydroxide at room temperature. The residue was purified by flash chromatography eluent: petroleum ether: ethyl acetate = 10: 1) to afford BA-06 as a white solid with a yield of 69%. ^1^H NMR (600 MHz, Chloroform-*d*) *δ* 7.85 (dd, *J* = 8.3, 2.7 Hz, 1H), 7.52 (d, *J* = 1.6 Hz, 1H), 7.38 (ddd, *J* = 9.9, 7.3, 2.7 Hz, 1H), 7.30–7.26 (m, 1H), 4.73–4.56 (m, 2H), 2.97 (td, *J* = 10.8, 5.1 Hz, 1H), 2.62 (dd, *J* = 15.8, 1.4 Hz, 1H), 2.27 (dt, *J* = 12.9, 3.3 Hz, 1H), 2.20 (td, *J* = 12.3, 3.6 Hz, 1H), 2.03–1.91 (m, 2H), 1.89–1.82 (m, 1H), 1.76–1.68 (m, 1H), 1.67 (s, 3H), 1.61 (t, *J* = 11.4 Hz, 1H), 1.51–1.44 (m, 3H), 1.42 (tt, *J* = 9.1, 4.9 Hz, 5H), 1.38–1.34 (m, 1H), 1.32 (d, *J* = 16.0 Hz, 1H), 1.27 (d, *J* = 17.8 Hz, 2H), 1.21 (ddt, *J* = 10.7, 8.3, 3.6 Hz, 2H), 1.18 (s, 3H), 1.13 (s, 3H), 0.97 (s, 3H), 0.94 (s, 3H), 0.79 (s, 3H).13C NMR (151 MHz, CDCl3) *δ* 207.58, 181.81, 150.46, 136.68, 132.88, 132.57, 132.52, 130.94, 128.86, 120.87, 120.73, 112.77, 112.59, 109.66, 77.25, 77.04, 76.83, 65.60, 56.38, 53.36, 49.08, 48.31, 46.81, 45.93, 43.21, 42.50, 40.55, 38.37, 37.01, 36.97, 33.16, 32.02, 30.58, 30.54, 29.62, 28.69, 25.42, 22.41, 21.44, 20.11, 19.42, 19.20, 15.70, 15.54, 14.57, 13.75. HRMS (ESI): C_37_H_48_FNNaO_5_ (628.3409) [M + Na]^+^ = 628.3409.

**BA-07** *(1R,3aS,5aR,5bR,7aR,11aR,11bR,13aR,13bR)-10-((E)-2-fluorobenzylidene)-5a,5b,8,8,11a-pentamethyl-9-oxo-1-(prop-1-en-2-yl)icosahydro-3aH-cyclopenta[a]chrysene-3a-carboxylic acid* (BA-07, C_37_H_49_FO_3_). According to the general procedure, derivative BA-07 was prepared by Claisen Schmidt condensation of intermediate BA-O with 2-fluorobenzaldehyde in the presence of ethanolic potassium hydroxide at room temperature. The residue was purified by flash chromatography (eluent: petroleum ether: ethyl acetate = 10: 1) to afford BA-07 as a white solid with a yield of 57%. ^1^H NMR (600 MHz, Chloroform-*d*) *δ* 7.59–7.55 (m, 1H), 7.32 (dddd, *J* = 12.9, 7.3, 6.1, 1.8 Hz, 2H), 7.17 (td, *J* = 7.5, 1.1 Hz, 1H), 7.12–7.06 (m, 1H), 5.00–4.54 (m, 2H), 3.00 (td, *J* = 10.8, 4.9 Hz, 1H), 2.91 (d, *J* = 16.1 Hz, 1H), 2.29 (dt, *J* = 13.0, 3.3 Hz, 1H), 2.23 (td, *J* = 12.3, 3.6 Hz, 1H), 2.11 (dd, *J* = 16.5, 3.1 Hz, 1H), 1.99 (dq, *J* = 11.9, 9.2, 8.0 Hz, 2H), 1.71 (s, 4H), 1.65 (t, *J* = 11.4 Hz, 1H), 1.52 (ddd, *J* = 18.2, 8.9, 4.1 Hz, 2H), 1.47 (d, *J* = 3.4 Hz, 2H), 1.46–1.39 (m, 6H), 1.28–1.21 (m, 2H), 1.14 (s, 3H), 1.13 (s, 3H), 1.08 (dd, *J* = 13.0, 4.4 Hz, 1H), 1.01 (s, 3H), 0.96 (s, 4H), 0.79 (s, 3H). ^13^C NMR (151 MHz, CDCl3) *δ* 207.69, 181.76, 160.04, 150.53, 109.65, 56.41, 53.07, 49.14, 48.36, 46.82, 45.41, 43.97, 42.51, 40.54, 38.42, 37.01, 36.64, 33.12, 32.05, 30.57, 29.66, 29.19, 25.55, 22.44, 21.53, 20.27, 19.46, 19.19, 15.77, 15.52, 14.60, 13.74. HRMS (ESI): C_37_H_49_FNaO_3_ (583.3558) [M + Na]^+^ = 583.3558.

**BA-08** *(1R,3aS,5aR,5bR,7aR,11aR,11bR,13aR,13bR)-10-((E)-3-bromobenzylidene)-5a,5b,8,8,11a-pentamethyl-9-oxo-1-(prop-1-en-2-yl)icosahydro-3aH-cyclopenta[a]chrysene-3a-carboxylic acid* (BA-08, C_37_H_49_BrO_3_). According to the general procedure, derivative BA-08 was prepared by Claisen Schmidt condensation of intermediate BA-O with 3-bromobenzaldehyde in the presence of ethanolic potassium hydroxide at room temperature. The residue was purified by flash chromatography (eluent: petroleum ether: ethyl acetate = 10: 1) to afford BA-08 as a white solid with a yield of 85%. ^1^H NMR (600 MHz, Chloroform-*d*) *δ* 7.51 (d, *J* = 1.8 Hz, 1H), 7.45 (dt, *J* = 7.9, 1.6 Hz, 1H), 7.39–7.37 (m, 1H), 7.34–7.31 (m, 1H), 7.28 (t, *J* = 7.8 Hz, 1H), 4.95–4.54 (m, 2H), 3.14–2.93 (m, 2H), 2.34–2.20 (m, 2H), 2.18–2.11 (m, 1H), 2.04–1.91 (m, 2H), 1.79–1.61 (m, 5H), 1.56–1.40 (m, 11H), 1.31–1.22 (m, 3H), 1.13 (s, 3H), 1.12 (s, 3H), 1.02 (s, 3H), 0.97 (s, 3H), 0.79 (s, 3H). ^13^C NMR (151 MHz, CDCl3) *δ* 208.00, 181.84, 181.78, 167.75, 150.42, 138.10, 135.66, 133.22, 132.32, 131.25, 130.93, 129.95, 128.86, 128.09, 122.55, 109.75, 77.25, 77.03, 76.82, 65.59, 56.43, 52.91, 49.15, 48.36, 46.84, 45.31, 44.13, 42.52, 40.64, 40.54, 38.42, 37.02, 36.62, 34.99, 34.88, 33.04, 32.05, 31.52, 31.45, 30.61, 30.58, 30.33, 30.20, 30.15, 29.71, 29.69, 29.36, 25.53, 22.36, 21.63, 20.33, 19.50, 19.20, 15.82, 15.50, 14.63, 13.75, 1.05. HRMS (ESI): C_37_H_50_^79^BrO_3_ (621.2938) [M + H]^+^ = 621.2938, C_37_H_50_^81^BrO_3_ (623.2917) [M + H]^+^ = 623.2919.

**BA-09** *(1R,3aS,5aR,5bR,7aR,11aR,11bR,13aR,13bR)-10-((E)-3-methoxybenzylidene-5a,5b,8,8,11a-pentamethyl-9-oxo-1-(prop-1-en-2-yl)icosahydro-3aH-cyclopenta[a]chrysene-3a-carboxylic acid* (BA-09, C_38_H_52_O_4_). According to the general procedure, derivative BA-09 was prepared by Claisen Schmidt condensation of intermediate BA-O with 3-methoxybenzaldehyde in the presence of ethanolic potassium hydroxide at room temperature. The residue was purified by flash chromatography (eluent: petroleum ether: ethyl acetate = 10: 1) to afford BA-09 as a white solid with a yield of 87%. ^1^H NMR (600 MHz, Chloroform-*d*) *δ* 7.45 (dd, *J* = 2.8, 1.5 Hz, 1H), 7.33 (t, *J* = 7.9 Hz, 1H), 7.01 (d, *J* = 7.7 Hz, 1H), 6.93 (t, *J* = 2.0 Hz, 1H), 6.88 (dd, *J* = 8.1, 2.6 Hz, 1H), 4.84–4.55 (m, 2H), 3.83 (s, 3H), 3.17–2.96 (m, 2H), 2.32–2.21 (m, 2H), 2.21–2.16 (m, 1H), 2.05–1.96 (m, 2H), 1.69 (d, *J* = 34.9 Hz, 5H), 1.57–1.38 (m, 12H), 1.27 (ddt, *J* = 16.6, 10.9, 3.8 Hz, 2H), 1.14 (s, 3H), 1.12 (s, 3H), 1.02 (s, 3H), 0.97 (s, 3H), 0.79 (s, 3H). ^13^C NMR (151 MHz, CDCl_3_) *δ* 208.24, 181.98, 159.43, 150.46, 137.32, 137.29, 134.55, 129.42, 122.39, 116.03, 113.97, 109.71, 56.44, 55.28, 52.86, 49.16, 48.39, 46.84, 45.22, 44.35, 42.50, 40.52, 38.44, 37.02, 36.52, 33.06, 32.06, 30.60, 29.68, 29.42, 25.56, 22.36, 21.63, 20.34, 19.48, 15.81, 15.49, 14.62. HRMS (ESI): C_38_H_53_O_4_ (573.3938) [M + H]^+^ = 573.3938.

**BA-10** *(1R,3aS,5aR,5bR,7aR,11aR,11bR,13aR,13bR)-10-(E)-4-fluorobenzylidene-5a,5b,8,8,11a-pentamethyl-9-oxo-1-(prop-1-en-2-yl)icosahydro-3aH-cyclopenta[a]chrysene-3a-carboxylic acid* (BA-10, C_37_H_49_FO_3_). According to the general procedure, derivative BA-10 was prepared by Claisen Schmidt condensation of intermediate BA-O with 4-fluorobenzaldehyde in the presence of ethanolic potassium hydroxide at room temperature. The residue was purified by flash chromatography (eluent: petroleum ether: ethyl acetate = 10: 1) to afford BA-10 as a white solid with a yield of 91%. ^1^H NMR (600 MHz, Chloroform-*d*) *δ* 7.45 (t, *J* = 2.0 Hz, 1H), 7.42–7.38 (m, 2H), 7.10 (t, *J* = 8.7 Hz, 2H), 4.79–4.62 (m, 2H), 3.07–2.96 (m, 2H), 2.33–2.22 (m, 2H), 2.21–2.16 (m, 1H), 2.07–1.95 (m, 2H), 1.70 (d, *J* = 34.3 Hz, 5H), 1.58–1.39 (m, 10H), 1.34–1.24 (m, 3H), 1.13 (d, *J* = 13.8 Hz, 7H), 1.03 (s, 3H), 0.97 (s, 3H), 0.78 (s, 3H). ^13^C NMR (151 MHz, CDCl_3_) *δ* 208.09, 182.06, 161.70, 150.53, 136.22, 133.82, 132.21, 132.16, 132.09, 115.66, 115.52, 109.69, 56.44, 52.76, 49.16, 48.45, 46.83, 45.15, 44.40, 42.51, 40.52, 38.45, 37.02, 36.48, 33.04, 32.05, 30.61, 29.67, 29.48, 25.61, 22.33, 21.66, 20.34, 19.51, 15.81, 15.47, 14.61. HRMS (ESI): C_37_H_49_FNaO_3_ (583.3558) [M + Na]^+^ = 583.3558.

**BA-11** *(1R,3aS,5aR,5bR,7aR,11aR,11bR,13aR,13bR)-5a,5b,8,8,11a-pentamethyl-9-oxo-1-(prop-1-en-2-yl)-10-((E)-3-(trifluoromethyl)benzylidene)icosahydro-3aH-cyclopenta[a]chrysene-3a-carboxylic acid* (BA-11, C_38_H_49_F_3_O_3_). According to the general procedure, derivative BA-11 was prepared by Claisen Schmidt condensation of intermediate BA-O with 3-(trifluoromethyl) benzaldehyde in the presence of ethanolic potassium hydroxide at room temperature. The residue was purified by flash chromatography (eluent: petroleum ether: ethyl acetate = 10: 1) to afford BA-11 as a white solid with a yield of 83%. ^1^H NMR (600 MHz, Chloroform-*d*) *δ* 7.62 (s, 1H), 7.60–7.56 (m, 2H), 7.56–7.51 (m, 1H), 7.46 (d, *J* = 2.4 Hz, 1H), 4.90–4.61 (m, 2H), 3.23–2.83 (m, 2H), 2.34–2.28 (m, 1H), 2.25 (td, *J* = 12.3, 3.6 Hz, 1H), 2.19 (dd, *J* = 16.2, 3.0 Hz, 1H), 2.00 (dq, *J* = 12.0, 9.2, 8.0 Hz, 2H), 1.77–1.62 (m, 5H), 1.56–1.39 (m, 10H), 1.30–1.23 (m, 3H), 1.14 (d, *J* = 6.2 Hz, 7H), 1.02 (s, 3H), 0.97 (s, 3H), 0.80 (s, 3H). ^13^C NMR (151 MHz, CDCl_3_) *δ* 207.94, 181.90, 150.39, 136.72, 135.99, 135.51, 132.50, 130.84, 128.95, 127.18, 124.83, 123.03, 109.74, 56.43, 52.92, 49.13, 48.42, 46.83, 45.35, 44.16, 42.52, 40.54, 38.43, 37.01, 36.62, 33.05, 32.04, 30.59, 29.68, 29.32, 25.49, 22.36, 21.62, 20.31, 19.46, 15.84, 15.49, 14.63. HRMS (ESI): C_38_H_50_F_3_O_3_ (611.3707) [M + H]^+^ = 611.3707.

**BA-12** (1R,3aS,5aR,5bR,7aR,11aR,11bR,13aR,13bR)-10-((E)-3-chlorobenzylidene)-5a,5b,8,8,11a-pentamethyl-9-oxo-1-(prop-1-en-2-yl)icosahydro-3aH-cyclopenta[a]chrysene-3a-carboxylic acid (BA-12, C_37_H_49_ClO_3_). According to the general procedure, derivative BA-12 was prepared by Claisen Schmidt condensation of intermediate 01 with 3-chlorobenzaldehyde in the presence of ethanolic potassium hydroxide at room temperature. The residue was purified by flash chromatography (eluent: petroleum ether: ethyl acetate = 10: 1) to afford BA-12 as a white solid with a yield of 79%. ^1^H NMR (600 MHz, Chloroform-*d*) *δ* 7.45–7.37 (m, 1H), 7.37–7.32 (m, 2H), 7.31–7.26 (m, 2H), 4.79–4.58 (m, 2H), 3.06–2.87 (m, 2H), 2.33–2.21 (m, 2H), 2.17 (dd, *J* = 16.4, 3.1 Hz, 1H), 2.06–1.95 (m, 2H), 1.69 (d, *J* = 34.2 Hz, 5H), 1.56–1.39 (m, 12H), 1.33–1.22 (m, 2H), 1.14 (s, 3H), 1.12 (s, 3H), 1.03 (s, 3H), 0.97 (s, 3H), 0.79 (s, 3H). ^13^C NMR (151 MHz, CDCl_3_) *δ* 208.03, 181.93, 150.40, 137.79, 135.77, 135.58, 134.35, 130.20, 129.69, 128.36, 127.81, 109.74, 56.44, 52.89, 49.14, 48.34, 46.83, 45.29, 44.15, 42.51, 40.52, 38.41, 37.01, 36.60, 33.02, 32.05, 30.60, 29.68, 29.36, 25.52, 22.35, 21.62, 20.32, 19.48, 15.81, 15.49, 14.62. HRMS (ESI): C_37_H_50_ClO_3_ (577.3443) [M + H]^+^ = 577.3443.

**BA-13** *(1R,3aS,5aR,5bR,7aR,11aR,11bR,13aR,13bR)-10-((E)-4-methoxybenzylidene)-5a,5b,8,8,11a-pentamethyl-9-oxo-1-(prop-1-en-2-yl)icosahydro-3aH-cyclopenta[a]chrysene-3a-carboxylic acid* (BA-13, C_38_H_52_O_4_). According to the general procedure, derivative BA-13 was prepared by Claisen Schmidt condensation of intermediate BA-O with 4-methoxybenzaldehyde in the presence of ethanolic potassium hydroxide at room temperature. The residue was purified by flash chromatography (eluent: petroleum ether: ethyl acetate = 10: 1) to afford BA-13 as a white solid with a yield of 80%. ^1^H NMR (600 MHz, Chloroform-*d*) *δ* 7.47 (t, *J* = 2.0 Hz, 1H), 7.44–7.36 (m, 2H), 6.97–6.92 (m, 2H), 4.81–4.60 (m, 2H), 3.85 (s, 3H), 3.11–2.94 (m, 2H), 2.33–2.23 (m, 2H), 2.23–2.18 (m, 1H), 2.07–1.96 (m, 2H), 1.71 (d, *J* = 35.7 Hz, 5H), 1.58–1.41 (m, 11H), 1.36–1.24 (m, 4H), 1.14 (s, 3H), 1.11 (s, 3H), 1.03 (s, 3H), 0.98 (s, 3H), 0.79 (s, 3H). ^13^C NMR (151 MHz, CDCl_3_) *δ* 208.12, 181.49, 159.82, 150.63, 137.29, 132.23, 132.23, 131.96, 128.67, 114.00, 114.00, 109.62, 56.43, 55.32, 52.66, 49.18, 48.51, 46.83, 45.01, 44.67, 42.51, 40.52, 38.46, 37.02, 36.40, 33.07, 32.06, 30.63, 29.69, 29.62, 25.66, 22.32, 21.68, 20.38, 19.54, 15.86, 15.48, 14.63. HRMS (ESI): C_38_H_53_O_4_ (573.3938) [M + H]^+^ = 573.3938.

### Synthesis of BA Derivative BA-14∼BA-22

**BA-14** *methyl (1R,3aS,5aR,5bR,7aR,11aR,11bR,13aR,13bR)-10-((E)-5-methoxy-2-nitrobenzylidene)-5a,5b,8,8,11a-pentamethyl-9-oxo-1-(prop-1-en-2-yl)icosahydro-3aH-cyclopenta[a]chrysene-3a-carboxylate* (BA-14, C_39_H_53_NO_6_). According to the general procedure, derivative BA-14 was prepared by Claisen Schmidt condensation of intermediate BA-O-Me with 5-methoxy-2-nitrobenzaldehyde in the presence of ethanolic potassium hydroxide at room temperature. The residue was purified by flash chromatography (eluent: petroleum ether: ethyl acetate = 10: 1) to afford BA-14 as a white solid with a yield of 66%. ^1^H NMR (600 MHz, Chloroform-*d*) *δ* 8.20 (d, *J* = 9.1 Hz, 1H), 7.59 (d, *J* = 2.6 Hz, 1H), 6.93 (dd, *J* = 9.2, 2.8 Hz, 1H), 6.67 (d, *J* = 2.7 Hz, 1H), 4.84–4.48 (m, 2H), 3.91 (s, 3H), 3.66 (s, 3H), 2.97 (td, *J* = 10.7, 4.5 Hz, 1H), 2.63 (dd, *J* = 15.6, 1.4 Hz, 1H), 2.29–2.13 (m, 2H), 1.92–1.80 (m, 3H), 1.69–1.55 (m, 5H), 1.49–1.31 (m, 11H), 1.25 (s, 3H), 1.18 (s, 3H), 1.13 (s, 3H), 0.95 (s, 3H), 0.92 (s, 3H), 0.81 (s, 3H). ^13^C NMR (151 MHz, CDCl_3_) *δ* 207.93, 176.58, 163.16, 150.81, 140.89, 135.64, 135.29, 134.86, 127.63, 115.74, 113.34, 109.43, 56.55, 56.06, 53.45, 51.33, 49.31, 48.32, 46.87, 45.99, 43.03, 42.46, 40.54, 38.23, 36.99, 36.90, 33.20, 32.03, 30.62, 29.71, 29.61, 28.62, 25.50, 22.44, 21.46, 20.15, 19.45, 15.73, 15.51, 14.59. HRMS (ESI): C_39_H_53_NNaO_6_ (654.3765) [M + Na]^+^ = 654.3765.

**BA-15** *methyl (1R,3aS,5aR,5bR,7aR,11aR,11bR,13aR,13bR)-10-((E)-5-fluoro-2-nitrobenzylidene)-5a,5b,8,8,11a-pentamethyl-9-oxo-1-(prop-1-en-2-yl)icosahydro-3aH-cyclopenta[a]chrysene-3a-carboxylate* (BA-15, C_38_H_50_FNO_5_). According to the general procedure, derivative BA-15 was prepared by Claisen Schmidt condensation of intermediate BA-O-Me with 5-fluoro-2-nitrobenzaldehyde in the presence of ethanolic potassium hydroxide at room temperature. The residue was purified by flash chromatography (eluent: petroleum ether: ethyl acetate = 10: 1) to afford BA-15 as a white solid with a yield of 71%. ^1^H NMR (600 MHz, Chloroform-*d*) *δ* 8.18 (d, *J* = 9.1 Hz, 1H), 7.58 (d, *J* = 2.5 Hz, 1H), 6.91 (dd, *J* = 9.2, 2.8 Hz, 1H), 6.64 (d, *J* = 2.7 Hz, 1H), 4.72–4.56 (m, 2H), 3.66 (s, 3H), 2.97 (td, *J* = 10.6, 4.5 Hz, 1H), 2.62 (dd, *J* = 15.7, 1.4 Hz, 1H), 2.25–2.18 (m, 2H), 1.89–1.86 (m, 1H), 1.84–1.78 (m, 1H), 1.66 (s, 3H), 1.67–1.53 (m, 2H), 1.47 (t, *J* = 7.0 Hz, 4H), 1.44–1.31 (m, 8H), 1.29–1.21 (m, 3H), 1.17 (s, 3H), 1.13 (s, 3H), 1.02 (d, *J* = 4.6 Hz, 1H), 0.95 (s, 3H), 0.92 (s, 3H), 0.80 (s, 3H). ^13^C NMR (151 MHz, CDCl_3_) *δ* 207.96, 176.58, 162.66, 150.86, 140.64, 135.52, 135.33, 135.03, 127.59, 115.88, 113.93, 109.40, 56.55, 53.44, 51.33, 49.31, 48.32, 46.86, 45.97, 43.04, 42.46, 40.54, 38.24, 36.98, 36.90, 33.20, 32.03, 30.62, 29.61, 28.63, 25.51, 22.42, 21.47, 20.15, 19.45, 15.72, 15.51, 14.56.

**BA-16** *methyl (1R,3aS,5aR,5bR,7aR,11aR,11bR,13aR,13bR)-10-((E)-3-fluorobenzylidene)-5a,5b,8,8,11a-pentamethyl-9-oxo-1-(prop-1-en-2-yl)icosahydro-3aH-cyclopenta[a]chrysene-3a-carboxylate* (BA-16, C_38_H_51_FO_3_). According to the general procedure, derivative BA-16 was prepared by Claisen Schmidt condensation of intermediate BA-O-Me with 3-fluorobenzaldehyde in the presence of ethanolic potassium hydroxide at room temperature. The residue was purified by flash chromatography (eluent: petroleum ether: ethyl acetate = 10: 1) to afford BA-16 as a white solid with a yield of 76%. ^1^H NMR (600 MHz, Chloroform-*d*) *δ* 7.45–7.39 (m, 1H), 7.37 (td, *J* = 8.0, 6.0 Hz, 1H), 7.17 (d, *J* = 7.7 Hz, 1H), 7.10 (dt, *J* = 10.1, 2.1 Hz, 1H), 7.02 (td, *J* = 8.4, 2.6 Hz, 1H), 4.93–4.58 (m, 2H), 3.67 (s, 3H), 3.04–2.93 (m, 2H), 2.31–2.21 (m, 2H), 2.18 (dd, *J* = 16.1, 3.3 Hz, 1H), 1.96–1.84 (m, 2H), 1.72 (s, 4H), 1.63 (t, *J* = 11.4 Hz, 1H), 1.52–1.34 (m, 11H), 1.33–1.18 (m, 2H), 1.13 (d, *J* = 18.7 Hz, 7H), 1.01 (s, 3H), 0.95 (s, 3H), 0.79 (s, 3H). ^13^C NMR (151 MHz, CDCl_3_) *δ* 208.12, 176.62, 161.79, 150.60, 138.11, 135.85, 135.49, 129.93, 126.04, 116.57, 115.22, 109.65, 56.58, 52.89, 51.34, 49.38, 48.44, 46.91, 45.27, 44.31, 42.48, 40.52, 38.29, 36.94, 36.56, 33.06, 32.08, 30.65, 29.65, 29.42, 25.60, 22.38, 21.70, 20.38, 19.49, 15.82, 15.41, 14.63. HRMS (ESI): C_38_H_51_FNaO_3_ (597.3714) [M + Na]^+^ = 597.3714.

**BA-17** *methyl (1R,3aS,5aR,5bR,7aR,11aR,11bR,13aR,13bR)-10-((E)-2-fluorobenzylidene)-5a,5b,8,8,11a-pentamethyl-9-oxo-1-(prop-1-en-2-yl)icosahydro-3aH-cyclopenta[a]chrysene-3a-carboxylate* (BA-17, C_38_H_51_FO_3_). According to the general procedure, derivative BA-17 was prepared by Claisen Schmidt condensation of intermediate BA-O-Me with 2-fluorobenzaldehyde in the presence of ethanolic potassium hydroxide at room temperature. The residue was purified by flash chromatography (eluent: petroleum ether: ethyl acetate = 10: 1) to afford BA-17 as a white solid with a yield of 75%. ^1^H NMR (600 MHz, Chloroform-*d*) *δ* 7.56 (t, *J* = 1.9 Hz, 1H), 7.32 (dtd, *J* = 12.9, 7.6, 1.8 Hz, 2H), 7.17 (td, *J* = 7.6, 1.2 Hz, 1H), 7.09 (ddd, *J* = 9.7, 8.2, 1.1 Hz, 1H), 4.82–4.52 (m, 2H), 3.67 (s, 3H), 3.05–2.95 (m, 1H), 2.91 (d, *J* = 16.3 Hz, 1H), 2.31–2.21 (m, 2H), 2.10 (dt, *J* = 16.0, 2.2 Hz, 1H), 1.90 (dddd, *J* = 13.7, 10.7, 7.5, 3.6 Hz, 2H), 1.70 (s, 4H), 1.62 (t, *J* = 11.4 Hz, 1H), 1.50–1.33 (m, 12H), 1.31–1.16 (m, 2H), 1.14 (d, *J* = 2.2 Hz, 6H), 1.00 (s, 3H), 0.95 (s, 3H), 0.80 (s, 3H). ^13^C NMR (151 MHz, CDCl_3_) *δ* 207.73, 176.61, 160.04, 150.76, 136.39, 130.34, 130.05, 129.84, 123.92, 123.82, 115.69, 109.52, 56.58, 53.13, 51.34, 49.38, 48.45, 46.89, 45.44, 43.97, 42.48, 40.54, 38.30, 36.94, 36.66, 33.16, 32.07, 30.65, 29.65, 29.18, 25.60, 22.49, 21.59, 20.31, 19.49, 15.77, 15.47, 14.63. HRMS (ESI): C_38_H_51_FNaO_3_ (597.3714) [M + Na]^+^ = 597.3714.

**BA-18** *methyl (1R,3aS,5aR,5bR,7aR,11aR,11bR,13aR,13bR)-10-((E)-3-bromobenzylidene)-5a,5b,8,8,11a-pentamethyl-9-oxo-1-(prop-1-en-2-yl)icosahydro-3aH-cyclopenta[a]chrysene-3a-carboxylate* (**BA-18**, C_38_H_51_BrO_3_). According to the general procedure, derivative BA-18 was prepared by Claisen Schmidt condensation of intermediate BA-O-Me with 3-bromobenzaldehyde in the presence of ethanolic potassium hydroxide at room temperature. The residue was purified by flash chromatography (eluent: petroleum ether: ethyl acetate = 10: 1) to afford BA-18 as a white solid with a yield of 89%. ^1^H NMR (600 MHz, Chloroform-*d*) *δ* 7.51 (t, *J* = 1.8 Hz, 1H), 7.44 (dt, *J* = 7.9, 1.6 Hz, 1H), 7.37 (dd, *J* = 3.0, 1.5 Hz, 1H), 7.36–7.30 (m, 1H), 7.28 (t, *J* = 7.8 Hz, 1H), 4.84–4.57 (m, 2H), 3.67 (s, 3H), 2.99 (ddd, *J* = 19.9, 13.4, 3.1 Hz, 2H), 2.35–2.19 (m, 2H), 2.17–2.12 (m, 1H), 1.90 (ddt, *J* = 13.3, 10.2, 5.5 Hz, 2H), 1.77–1.57 (m, 6H), 1.51–1.35 (m, 10H), 1.31–1.16 (m, 3H), 1.14 (s, 3H), 1.12 (s, 3H), 1.01 (s, 3H), 0.95 (s, 3H), 0.78 (s, 3H). ^13^C NMR (151 MHz, CDCl_3_) *δ* 208.00, 176.61, 150.62, 138.11, 135.73, 135.58, 133.21, 131.22, 129.93, 128.05, 122.53, 109.59, 56.58, 52.94, 51.33, 49.36, 48.43, 46.88, 45.31, 44.12, 42.48, 40.52, 38.27, 36.92, 36.62, 33.06, 32.06, 30.65, 29.65, 29.34, 25.56, 22.39, 21.67, 20.35, 19.50, 15.81, 15.42, 14.64. HRMS (ESI): C_38_H_51_^79^BrNaO_3_ (657.2914) [M + Na]^+^ = 657.2914, C_38_H_51_^81^BrNaO_3_ (659.2893) [M + Na]^+^ = 659.2894.

**BA-19** *methyl (1R,3aS,5aR,5bR,7aR,11aR,11bR,13aR,13bR)-10-((E)-3-chlorobenzylidene)-5a,5b,8,8,11a-pentamethyl-9-oxo-1-(prop-1-en-2-yl)icosahydro-3aH-cyclopenta[a]chrysene-3a-carboxylate* (BA-19, C_38_H_51_ClO_3_). According to the general procedure, derivative BA-19 was prepared by Claisen Schmidt condensation of intermediate BA-O-Me with 3-chlorobenzaldehyde in the presence of ethanolic potassium hydroxide at room temperature. The residue was purified by flash chromatography (eluent: petroleum ether: ethyl acetate = 10: 1) to afford BA-19 as a white solid with a yield of 53%. ^1^H NMR (600 MHz, Chloroform-*d*) *δ* 7.38 (d, *J* = 2.6 Hz, 1H), 7.37–7.31 (m, 2H), 7.31–7.26 (m, 2H), 4.96–4.52 (m, 2H), 3.67 (s, 3H), 3.26–2.88 (m, 2H), 2.31–2.23 (m, 2H), 2.16 (dd, *J* = 16.3, 3.0 Hz, 1H), 1.99–1.86 (m, 2H), 1.80–1.54 (m, 6H), 1.51–1.34 (m, 11H), 1.32–1.19 (m, 3H), 1.14 (s, 3H), 1.12 (s, 3H), 1.01 (s, 3H), 0.95 (s, 3H), 0.78 (s, 3H). ^13^C NMR (151 MHz, CDCl_3_) *δ* 208.04, 176.62, 150.63, 137.82, 135.70, 135.68, 134.35, 130.21, 129.69, 128.34, 127.78, 109.61, 56.59, 52.95, 51.34, 49.38, 48.43, 46.90, 45.31, 44.16, 42.49, 40.53, 38.28, 36.93, 36.62, 33.07, 32.07, 30.66, 29.66, 29.36, 25.57, 22.40, 21.68, 20.36, 19.51, 15.81, 15.43, 14.64. HRMS (ESI): C_38_H_51_ClNaO_3_ (613.3419) [M + Na]^+^ = 613.3420.

**BA-20** *methyl (1R,3aS,5aR,5bR,7aR,11aR,11bR,13aR,13bR)-5a,5b,8,8,11a-pentamethyl-9-oxo-1-(prop-1-en-2-yl)-10-((E)-3-(trifluoromethyl)benzylidene)icosahydro-3aH-cyclopenta[a]chrysene-3a-carboxylate* (BA-20, C_39_H_51_F_3_O_3_). According to the general procedure, derivative BA-20 was prepared by Claisen Schmidt condensation of intermediate BA-O-Me with 3-(trifluoromethyl)benzaldehyde in the presence of ethanolic potassium hydroxide at room temperature. The residue was purified by flash chromatography (eluent: petroleum ether: ethyl acetate = 10: 1) to afford BA-20 as a white solid with a yield of 58%. ^1^H NMR (600 MHz, Chloroform-*d*) *δ* 7.62 (s, 1H), 7.59–7.56 (m, 2H), 7.55–7.50 (m, 1H), 7.46 (dd, *J* = 3.0, 1.5 Hz, 1H), 4.87–4.49 (m, 2H), 3.67 (s, 3H), 3.05–2.93 (m, 2H), 2.31–2.23 (m, 2H), 2.19–2.14 (m, 1H), 2.01–1.82 (m, 2H), 1.76–1.56 (m, 6H), 1.54–1.37 (m, 11H), 1.32–1.17 (m, 2H), 1.15 (s, 3H), 1.13 (s, 3H), 1.01 (s, 3H), 0.95 (s, 3H), 0.80 (s, 3H). ^13^C NMR (151 MHz, CDCl_3_) *δ* 207.96, 176.61, 150.60, 136.74, 136.08, 135.43, 132.47, 130.84, 128.94, 127.18, 124.80, 123.03, 109.59, 56.58, 52.97, 51.34, 49.36, 48.49, 46.89, 45.36, 44.16, 42.48, 40.53, 38.29, 36.92, 36.63, 33.08, 32.06, 30.65, 29.65, 29.31, 25.53, 22.40, 21.67, 20.34, 19.48, 15.84, 15.43. HRMS (ESI): C_39_H_51_F_3_NaO_3_ (647.3683) [M + Na]^+^ = 647.3685.

**BA-21** *methyl (1R,3aS,5aR,5bR,7aR,11aR,11bR,13aR,13bR)-10-((E)-4-methoxybenzylidene)-5a,5b,8,8,11a-pentamethyl-9-oxo-1-(prop-1-en-2-yl)icosahydro-3aH-cyclopenta[a]chrysene-3a-carboxylate* (BA-21, C_39_H_54_O_4_). According to the general procedure, derivative BA-21 was prepared by Claisen Schmidt condensation of intermediate BA-O-Me with 4-methoxybenzaldehyde in the presence of ethanolic potassium hydroxide at room temperature. The residue was purified by flash chromatography (eluent: petroleum ether: ethyl acetate = 10: 1) to afford BA-21 as a white solid with a yield of 77%. ^1^H NMR (600 MHz, Chloroform-*d*) *δ* 7.47 (t, *J* = 1.9 Hz, 1H), 7.32 (d, *J* = 7.9 Hz, 2H), 7.22 (d, *J* = 7.9 Hz, 2H), 4.80–4.61 (m, 2H), 3.67 (s, 3H), 3.11–2.96 (m, 2H), 2.38 (s, 3H), 2.30–2.23 (m, 2H), 2.24–2.17 (m, 1H), 1.96–1.82 (m, 2H), 1.73 (s, 3H), 1.64 (t, *J* = 11.4 Hz, 1H), 1.52–1.36 (m, 10H), 1.35–1.18 (m, 4H), 1.15 (s, 3H), 1.11 (s, 3H), 1.01 (s, 3H), 0.96 (s, 3H), 0.78 (s, 3H). ^13^C NMR (151 MHz, CDCl_3_) *δ* 208.09, 176.60, 150.71, 136.53, 136.43, 136.07, 132.86, 130.14, 129.41, 127.05, 124.80, 109.51, 56.56, 53.35, 51.33, 49.36, 48.44, 46.88, 45.74, 43.35, 42.47, 40.55, 38.27, 36.93, 36.87, 33.21, 32.06, 30.62, 29.64, 28.90, 25.54, 22.47, 21.52, 20.23, 19.46, 15.73, 15.50, 14.62.

**BA-22** *methyl (1R,3aS,5aR,5bR,7aR,11aR,11bR,13aR,13bR)-10-((E)-2-bromobenzylidene)-5a,5b,8,8,11a-pentamethyl-9-oxo-1-(prop-1-en-2-yl)icosahydro-3aH-cyclopenta[a]chrysene-3a-carboxylate* (BA-22, C_38_H_51_BrO_3_). According to the general procedure, derivative BA-22 was prepared by Claisen Schmidt condensation of intermediate BA-O-Me with 2-bromobenzaldehyde in the presence of ethanolic potassium hydroxide at room temperature. The residue was purified by flash chromatography (eluent: petroleum ether: ethyl acetate = 10: 1) to afford BA-22 as a white solid with a yield of 68%. ^1^H NMR (600 MHz, Chloroform-*d*) *δ* 7.61 (dd, *J* = 7.9, 1.2 Hz, 1H), 7.48 (d, *J* = 2.7 Hz, 1H), 7.33 (td, *J* = 7.5, 1.2 Hz, 1H), 7.24–7.19 (m, 1H), 7.17 (td, *J* = 7.7, 1.7 Hz, 1H), 5.09–4.41 (m, 2H), 3.66 (s, 3H), 3.05–2.94 (m, 1H), 2.82 (dd, *J* = 15.9, 1.5 Hz, 1H), 2.23 (td, *J* = 12.3, 3.4 Hz, 2H), 2.03–1.81 (m, 3H), 1.68 (s, 5H), 1.49–1.34 (m, 10H), 1.30–1.18 (m, 4H), 1.16 (s, 3H), 1.14 (s, 3H), 0.97 (s, 3H), 0.94 (s, 3H), 0.81 (s, 3H). ^13^C NMR (151 MHz, CDCl_3_) *δ* 208.09, 176.60, 150.71, 136.53, 136.43, 136.07, 132.86, 130.14, 129.41, 127.05, 124.80, 109.51, 56.56, 53.35, 51.33, 49.36, 48.44, 46.88, 45.74, 43.35, 42.47, 40.55, 38.27, 36.93, 36.87, 33.21, 32.06, 30.62, 29.64, 28.90, 25.54, 22.47, 21.52, 20.23, 19.46, 15.73, 15.50, 14.62. HRMS (ESI): C_38_H_51_^79^BrNaO_3_ (657.2914) [M + Na]^+^ = 657.2914, C_38_H_51_^81^BrNaO_3_ (659.2893) [M + Na]^+^ = 659.2894.

### HAase Inhibition Assay

The effect of BA derivatives on HAase activity was evaluated using a previous report with some modifications (Bahadir Acikara et al., 2019; He et al., 2021). Briefly, BA derivatives were prepared to 100 mM in DMSO, then diluted with DMSO yield concentrations ranging from 6.25 to 100 μg/mL. 5 μL test samples and 95 μL HAase protein (7.5 U/mL) were dissolved in sodium phosphate buffer (20 mM) containing 0.01% BSA (pH = 7.0) and incubated at 37°C for 10 min. Then HA (100 μl) to the mixture, followed incubation at 37°C for 45 min. Undigested HA was precipitated by adding the acidic albumin solution (1 ml; pH = 3.75) containing 0.1% BSA which dissolved in sodium acetate (24 mM) and acetic acid (79 mM) solution. After 10 min, absorbance was measured at a wavelength of 600 nm using a microplate reader (SpectraMax M2, Molecular Devices Corp., operated by SoftmaxPro v.4.6 software, Sunnyvale, CA, United States). The inhibition rate was calculated using following formula: Inhibition% = 1 − [OD (HA) − OD (sample)]/[OD (HA) − OD (HAase)]. The IC_50_ value of each sample was determined by analyzing its inhibition rates at different concentrations with a nonlinear regression algorithm by GraphPad Prism.

### Molecular Docking

The molecular docking analysis was conducted on the molecular modeling station provided by the Rhode Island IDeA Network of Biomedical Research Excellence (RI-INBRE) using the Autodock 4.2 and Autodock Tools base on the Lamarckian Genetic Algorithm (LGA). The structure of compound BA derivatives was generated by Chemdraw 3D (PerkinElmer Inc.; Waltham, MA, United States). And the crystal structure of collagenase protein (PDB ID: 2PE4) was retrieved in PDB format from the RCSB protein data bank (www.rcsb.org). The HAase protein was prepared by removing water molecules and adding force field parameters and their co-crystallized ligands were used for assigning the ligand-binding domain for the docking simulations. The optimized protein structure, construction of missing side chains and loops, binding site, and the binding score of the ligands were obtained on the basis of their free banding energy and hydrogen bond. The 2D diagrams of the protein-ligand interactions were visualized by Discovery Studio (DS; BIOVIA Corp.; San Diego, CA, United States).

### Cell Culture

Murine macrophage RAW264.7 cells were purchased from the American Type Culture Collection (ATCC; Rockville, MD, United States). The BV-2 murine microglial cells were a gift kindly provided by Dr. Grace Y. Sun (The University of Missouri at Columbia, MO, United States). Cells were cultured in Dulbecco’s Modified Eagle’s Medium (DMEM) supplemented with 10% (v/v) Fetal bovine serum (FBS) (Gibco, Life Technologies, Gaithersburg, MD, United States) and 1% (v/v) Penicillin/Streptomycin antibiotic solution (Gibco, Life Technologies, Grand Island, NY, United States) and placed in an incubator maintained at temperature 37°C under humidified atmospheric conditions consisting of 5% CO_2_. Human THP-1 monocytes were purchased from the ATCC. The cells were cultured in Roswell Park Memorial Institute (RPMI) 1640 medium supplemented with 10% (v/v) FBS and 1% (v/v) Penicillin/Streptomycin antibiotic solution, placed in an incubator maintained at a temperature of 37°C under humidified atmospheric conditions consisting of 5% CO_2_. THP-1 monocytes were differentiated by incubation with phorbol 12-myristate 13-acetate (PMA from Sigma Aldridge; 25 nM) for 48 h. Then PMA was removed and cells were cultured with PMA-free medium for another 24 h.

### Cell Viability

The cytotoxicity of each compound was determined in BV-2 and RAW264.7 cells with the MTT assay (Li et al., 2020). Briefly, cells were seeded in 96-well plates at 20,000 cells per well. Cells were allowed to adhere for 24 h. BA derivatives were prepared to 100 mM in DMSO, then diluted in serum-free media to yield concentrations of 10 µM. After the treatment of the cells with BA derivatives for 2 h, the cells were incubated in the absence or presence of LPS (1 μg/mL) for 24 h. Cellular viability was determined as a percentage of control (DMSO) by using a Cell Proliferation Kit II (Promega, Madison, WI, United States). XTT reagent (0.3 mg/mL) was then added to each well and incubated for 4 h at 37°C and an absorbance value was measured at 492 nm using a Spectramax M2 microplate reader (Hayon et al., 2003).

### NO Measurement

NO concentration was determined by the Griess assay according to a previous report (Ma et al., 2016), RAW264.7 and BV-2 cells (20,000 cells per well) were plated into 96-well plates. After an incubation period of 24 h, the cells were pre-treated with BA derivatives for 2 h and were co-treated with LPS (1 μg/mL) for 24 h. For the LMWHAFs-induced inflammation assay, THP-1 cells were pre-treated with BA derivatives for 2 h and then treated with LPS (1 μg/mL) and HA fragment (a hyaluronan oligosaccharide 6mer; 50 μg/mL) from Amsbio (Rockville, MD, United States; product code: CSR-11002) for 24 h (Campo et al., 2012; Scuruchi et al., 2016). Subsequently, the culture medium was mixed with the Griess reagent in a 1:1 ratio followed by a 15 min incubation in the dark. An absorbance value was measured at 540 nm using a Spectramax M2 microplate reader, and the NO concentration was calculated based on the standard NO curve.

### Measurement of IL-6 by ELISA Assay

To evaluate the effect of BA derivatives on LPS-induced IL-6 secretion, THP-1 monocytes were seeded at a density of 50,000 cells per well in a 48-well plate and differentiated with PMA (25 ng/mL), pre-treated with the BA derivatives at concentrations of 10 μM was performed for 6 h followed by stimulation with or without 1 μg/mL LPS for 12 h. The cell culture supernatant was collected for the measurement of IL-6. The levels of IL-6 were determined using specific ELISA kits (BioLegend, San Diego, CA, United States) (Liu et al., 2020).

## Results and Discussion

### Synthesis of BA Derivatives

**SCHEME 1:**
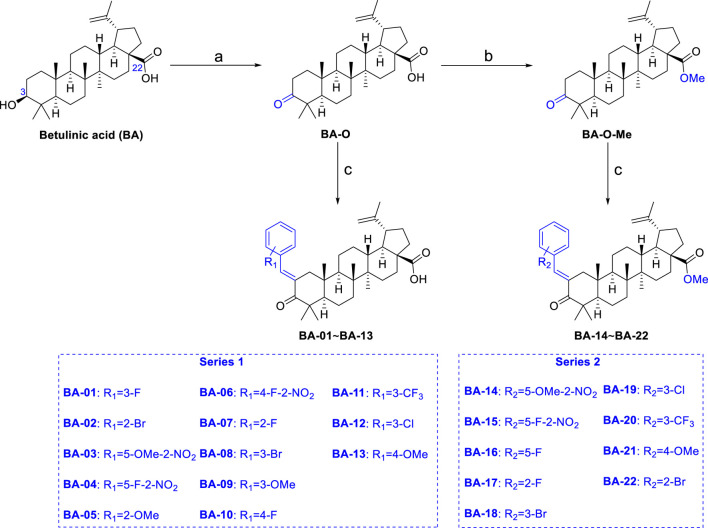
Chemical synthesis of two series of BA derivatives. Reagents and conditions: **(A)** Jones reagent, acetone, 0°C to r.t., 2 h, 92%; **(B)** CH_3_I, K_2_CO_3_, DMF, N_2_, r.t., overnight, 98%; **(C)** R-CHO, KOH, EtOH, r.t., 3 h, 53–91%.

A series of novel α,β-unsaturated BA ketene derivatives was synthesized using BA as the initial starting compound. The synthetic routes and chemical structures of two sets of BA derivatives including α,β-unsaturated ketene analogues (BA-O), and their methylated analogues (BA-O-Me) are shown in Scheme 1. The intermediate BA-O was synthesized by modifying the C-3 position of BA to obtain a carbonyl moiety with the Jones reagent, followed by converting it to the other intermediate BA-O-Me by modifying the -COOH group at the C-22 position via esterification with CH_3_I and K_2_CO_3_ in DMF. Next, two series of target derivatives were generated by Claisen Schmidt condensation at the C-3 position of the ketone intermediates BA-O and BA-O-Me to obtain derivatives BA-01∼BA-13 (Series 1) and BA-14∼BA-22 (Series 2), respectively. All compounds were purified by column chromatography with yields in a range between 53% and 91%, and their chemical structures were identified by a combination of spectroscopic analyses including ^1^H NMR, ^13^C NMR, and HRMS.

### Inhibition of BA Derivatives on HAase Activity

**FIGURE 1:**
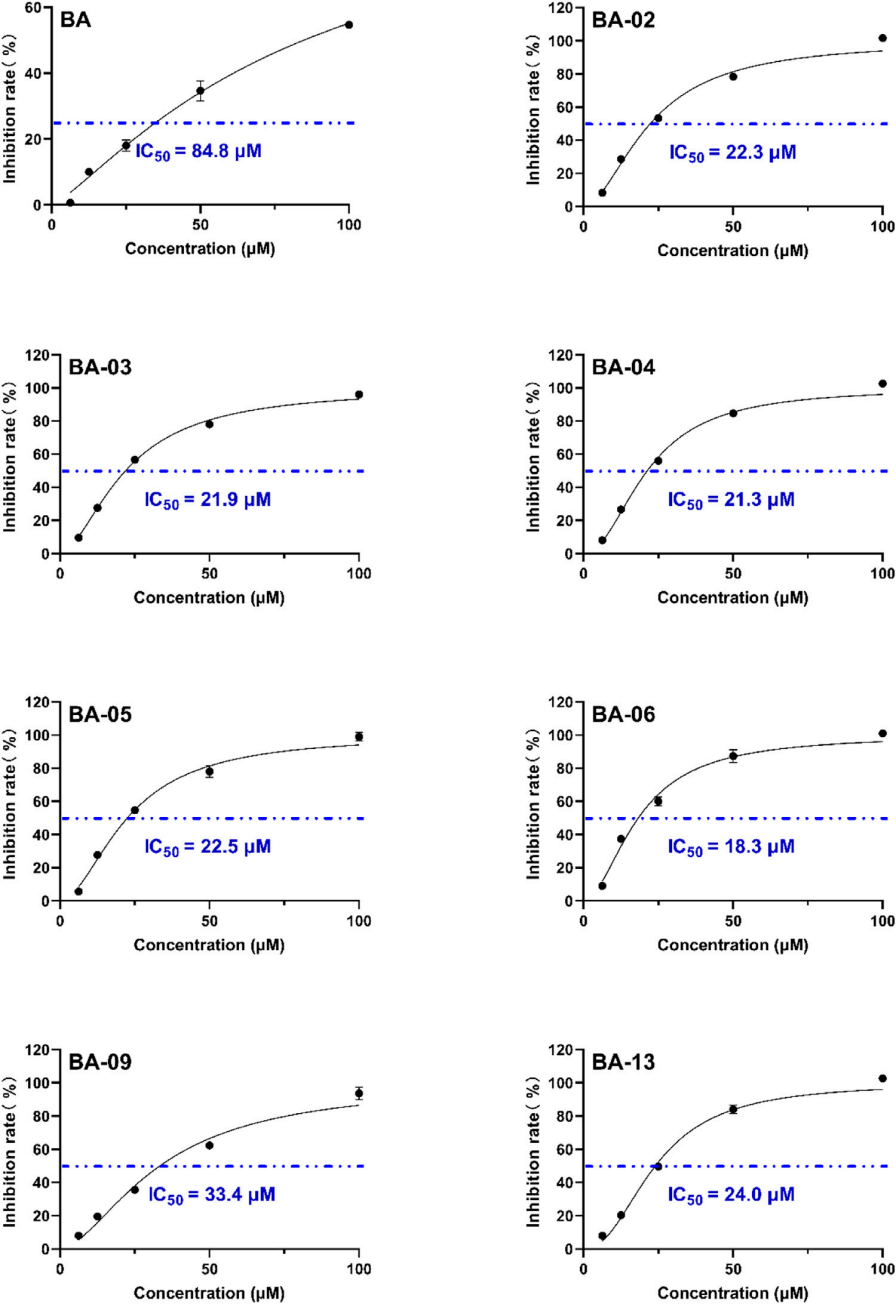
Effects of leading BA derivatives on the activity of HAase enzyme activity. The inhibition rates (%) of each compound were measured at five concentrations (1, 5, 25, 50, and 100 µM) and an inhibition curve of each compound was constructed to calculate the IC_50_ value by analyzing the inhibition rates at different concentrations with a nonlinear regression algorithm using GraphPad Prism.

We first evaluated the inhibitory effects of BA derivatives on HAase enzyme at a threshold concentration of 40 μm. Compounds of Series 1 (BA-01∼BA-13) showed anti-HAase activity with an inhibition rate of 21.3%–77.7% (Table 1). The BA-O derivatives including BA-01, BA-02, BA-03, BA-04, BA-05, BA-06, BA-09, and BA-13 showed the most potent inhibitory effect on HAase, which was superior to BA (inhibition of 44.1, 65.4, 46.5, 72.8, 68.1, 52.7, 42.4 and 77.7%, vs. 22.6%, respectively), whilst derivatives BA-14∼BA-22 (Series 2) had weaker anti-HAase activity (1.0%–14.9% inhibition, respectively). Next, the inhibitory effects of BA derivatives with promising enzyme inhibition activity were further evaluated by obtaining their IC_50_ inhibition value. The potency of BA-O derivatives is in an order of BA-02, BA-03, BA-04, BA-05, BA-06, BA-09, BA-13 with an IC_50_ value of 22.3, 21.9, 21.3, 22.5, 18.3, 33.4 and 24.0 µM, respectively, (Figure 1). Oleanolic acid (OA; a pentacyclic terpenoid) was used as a positive control showing an inhibitory effect on HAase by 31.7% at a concentration of 40 μM.

**TABLE 1:** The structure and functional groups of **BA** derivatives and their inhibition rate of HAase (at a threshold concentration of 40 µM).

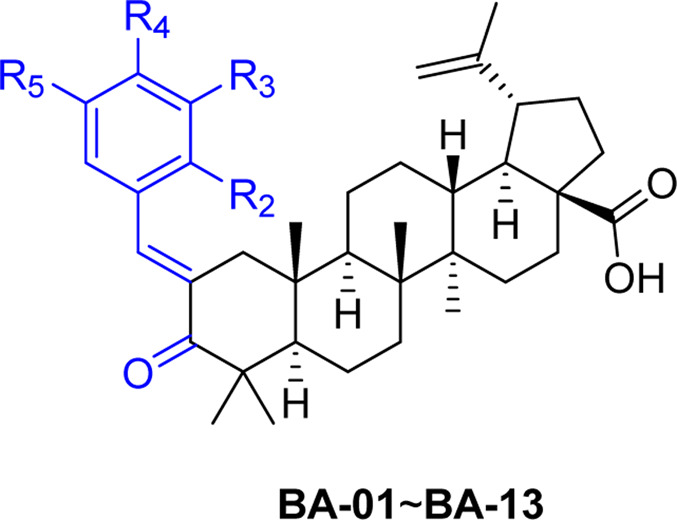						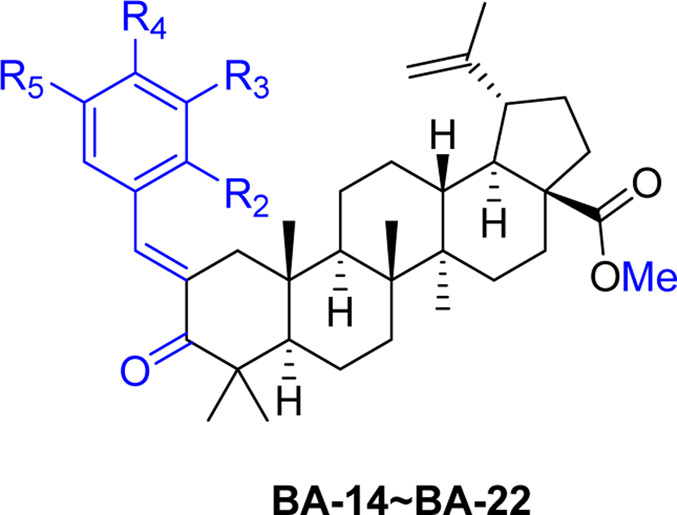					
**Cpd**	**R_2_=**	**R_3_=**	**R_4_=**	**R_5_=**	**%** ^**a**^	**Cpd**	**R_2_=**	**R_3_=**	**R_4_=**	**R_5_=**	**%** ^**a**^
**BA-01**	H	F	H	H	44.1 ± 2.7	**BA-14**	NO_2_	H	H	OMe	12.5 ± 3.4
**BA-02**	Br	H	H	H	65.4 ± 0.4	**BA-15**	NO_2_	H	H	F	14.9 ± 2.1
**BA-03**	NO_2_	H	H	OMe	46.5 ± 3.6	**BA-16**	H	H	H	F	1.0 ± 1.1
**BA-04**	NO_2_	H	H	F	72.8 ± 0.9	**BA-17**	F	H	H	H	4.9 ± 1.9
**BA-05**	OMe	H	H	H	68.1 ± 6.8	**BA-18**	H	Br	H	H	6.7 ± 2.8
**BA-06**	NO_2_	H	F	H	52.7 ± 1.4	**BA-19**	H	Cl	H	H	3.4 ± 2.5
**BA-07**	F	H	H	H	26.3 ± 2.8	**BA-20**	H	CF_3_	H	H	8.8 ± 1.4
**BA-08**	H	Br	H	H	35.3 ± 6.6	**BA-21**	H	H	OMe	H	7.5 ± 2.3
**BA-09**	H	OMe	H	H	42.4 ± 2.1	**BA-22**	Br	H	H	H	5.5 ± 1.0
**BA-10**	H	H	F	H	35.5 ± 2.5						
**BA-11**	H	CF_3_	H	H	31.6 ± 2.0						
**BA-12**	H	Cl	H	H	21.3 ± 1.1						
**BA-13**	H	H	OMe	H	77.7 ± 1.9						

a Inhibition rate against HAase of test compounds at 40 µM.

Some preliminary structure and activity relationship (SAR) can be observed based on the anti-HAase activity of BA derivatives. For instance, an introduction of functional groups including halogen, nitro, methoxy, and trifluoromethyl moiety at the phenyl ring increased the anti-HAase activity of BA-O compounds as compared to their BA skeleton (by up to 400%). Our preliminary SAR analysis suggested that the phenyl ring on the BA skeleton with electron-withdrawing groups (e.g., halogen, nitro, methoxy and trifluoromethyl) is critical for the anti-HAase activity. In addition, the numbers of electron-withdrawing groups on the benzene ring seemed to be important as multiple electron-withdrawing groups had stronger HAase inhibitory activities. For instance, fluorine in the para-position and a nitro group in the ortho position of the phenyl ring resulted in higher anti-HAase activity (IC_50_ = 18.3 μM) as compared to a BA-O derivative with a single bromo group in the ortho position of the phenyl ring (IC_50_ = 22.3 μM). Furthermore, induction of a methyl group *via* esterification with the -COOH group at position C-28 of BA resulted in the loss of anti-HAase activity of BA-O-Me derivatives. This may be attributed to the C-28-COOH group being critical for the maintenance of the anti-HAase activity given that this moiety can induce hydrophilic interaction with HAase enzyme. This SAR observation is in agreement with our previously reported study showing that the derivatives of OA (a triterpenoid with a carboxylic acid at the C-28 position) require the -COOH group intact to impart their HAase activity (He et al., 2021). In addition, this SAR pattern is also applicable to the triterpenoid ursolic acid (UA) and its derivatives where the carboxylic acid at the C-28 position is also essential for their inhibitory effects on HAase (Abdullah et al., 2016).

### Interactions Between BA Derivatives and HAase Protein

**FIGURE 2:**
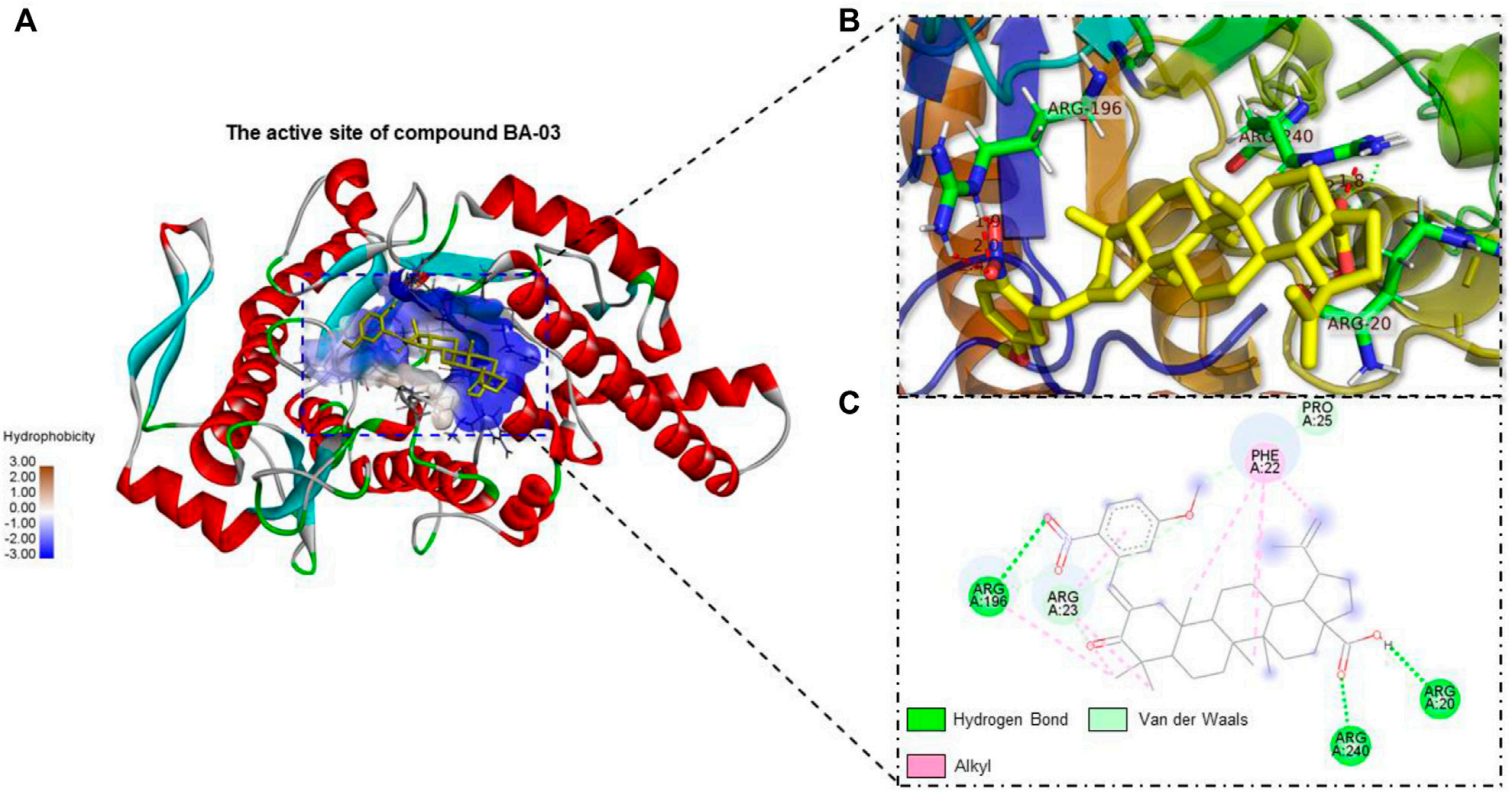
Molecule docking analysis of the interactions between HAase enzyme protein and BA derivatives. **(A)** Binding pocket of HAase with embedded compound BA-03; **(B)** The active site of most potent compound BA-03; **(C)** The molecular interactions of compound BA-03 and HAase showing formed molecular forces.

Interactions between HAase enzyme protein and BA-O based HAase inhibitors including BA-02∼BA-06, BA-09, BA-13 were investigated by molecular docking. The predicted protein-ligand complexes were evaluated on the basis of minimum free binding energy values (kcal/mol). Docking results showed that the aforementioned BA-O compounds had favorable binding energy ranging from −8.7 to −11.4 kcal/mol (Table 2). The predicted binding parameters were in agreement with data obtained from the enzyme inhibition assay. For instance, BA-03, which was the most active HAase inhibitor, had the lowest free binding energy (−11.4 kcal/mol) and binding constant (4.6 µM). As shown in Figure 2, BA-03 bound to a hydrophilic pocket on the HAase protein and interacted with several amino acid residues including Arg196, Arg240, and Arg20 via its -COOH group to form covalent bonding (i.e., hydrogen bond), which enhanced the stability of the ligand-protein complex. This conclusion is in agreement with our previously reported results showing that triterpenoid derivatives can bind to HAase protein via the formation of several molecular forces including hydrogen and alkyl bonds, which may contribute to their anti-HAase activity (He et al., 2021). Although data from the molecular docking experiments provide insights on possible binding modes of BA derivatives and HAase protein, further studies using biophysical methods (e.g., binding assays and the X-ray crystallography) are warranted to elucidate the binding property of BA-based ligands and HAase.

**TABLE 2:** Estimated free binding energy and inhibition constant of BA derivatives by molecular docking method.

Compounds	Banding energy (kcal/mol)	*K*_*i*_ (Inhibition constant; µM)
**BA-02**	−8.8	338.0
**BA-03**	−11.4	4.6
**BA-04**	−9.5	115.9
**BA-05**	−8.7	436.3
**BA-06**	−9.2	169.0
**BA-09**	−9.2	169.0
**BA-13**	−8.7	434.7

### Inhibitory Effects of BA Derivatives on NO Production in LPS-Stimulated BV2 Cells

**FIGURE 3:**
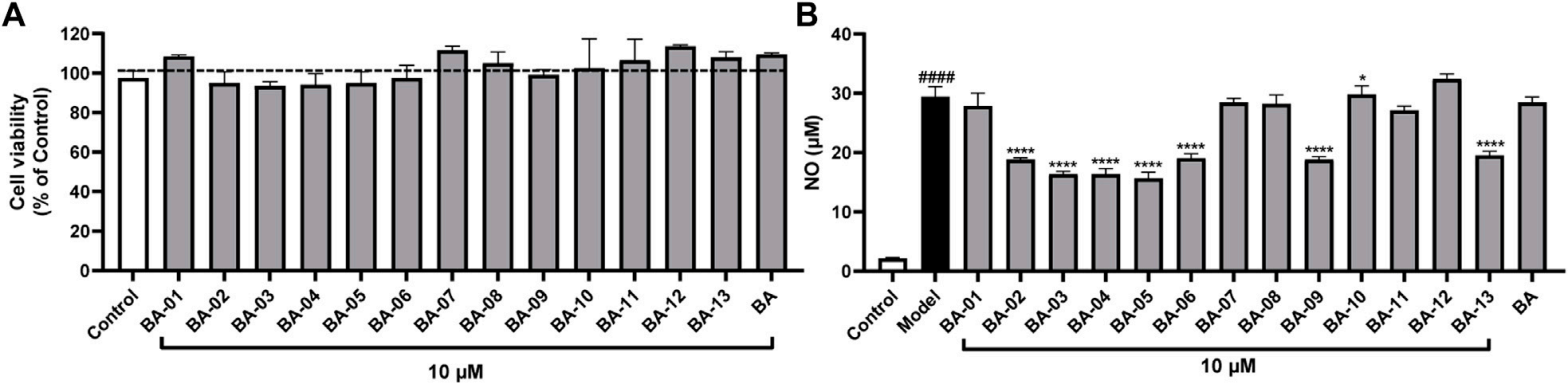
Effects of BA derivatives on the cell viability of BV-2 cells **(A)** and effects of BA derivatives on the NO production in BV2 cells with or without the stimulation of LPS. **(B)** Data presented as mean ± S.E. (*n* = 3). Significance was reported by analysis of variance (ANOVA) followed with Dunnett multiple comparison testing. Significance as compared to the control group *p* ≤ 0.0001 (^####^); or compared to the model (LPS-stimulated) group, *p* ≤ 0.05 (∗) and *p* ≤ 0.0001 (∗∗∗∗).

Given that the inhibition of HAase may result in less production of fragmented HAs, which is an inducing factor for inflammatory response, we further assessed whether BA based HAase inhibitors can exert anti-inflammatory effects. In this study, we first evaluated the anti-inflammatory activity of BA derivatives in LPS-stimulated murine microglial BV2 cells by measuring the production of nitrite (NO). Prior to conducting the anti-inflammatory assays, BV2 cells were treated with BA and its derivatives BA-01∼13 at a concentration of 10 μM. Compounds tested at this concentration did not show any cytotoxicity with cell viability of 109.5% and 93.5%–113.5%, respectively, (Figure 3A). BA derivatives were further evaluated at the nontoxic concentration (10 μM). As shown in Figure 3B, BA derivatives including BA-02∼BA-06, BA-09, BA-11, and BA-13 exerted anti-inflammatory effects by inhibiting the LPS-stimulated NO production by 35.1%–46.8%, 36.0%, 7.7%, and 33.6%, respectively. These BA derivatives showed superior NO inhibition effects as compared to their parent compound BA with a NO reduction of 3.4%.

**FIGURE 4:**
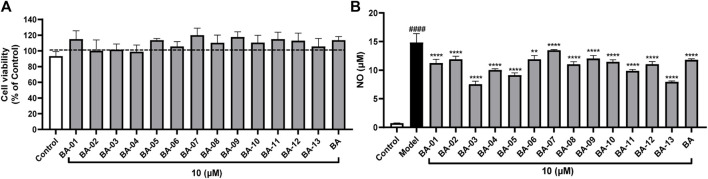
Effects of BA derivatives on the cell viability of RAW 264.7 cells **(A)** and effects of BA derivatives on the NO production in RAW 264.7 cells with or without the stimulation of LPS. **(B)** Data presented as mean ± S.E. (*n* = 3). Significance was reported by analysis of variance (ANOVA) followed with Dunnett multiple comparison testing. Significance as compared to the control group *p* ≤ 0.0001 (^####^); or compared to the model (LPS-stimulated) group, *p* ≤ 0.01 (∗∗) and *p* ≤ 0.0001 (∗∗∗∗).

Additionally, the NO inhibitory effects of BA and its derivatives were evaluated in murine macrophage RAW264.7 cells. All of the BA derivatives showed no cytotoxicity at a concentration of 10 μM with cell viability no less than 99.0% (Figure 4A). BA showed significant anti-inflammatory effects by decreasing the LPS-stimulated NO production by 20.4% (Figure 4B). BA derivatives including BA-03, BA-04, BA-05, BA-13, and BA-11 showed stronger anti-inflammatory effects as compared to BA with a reduction in NO levels of 42.9%, 32.3%, 38.5%, 46.4%, and 33.4%, respectively. Compounds BA-03 and BA-13 had the most potent activities by decreasing NO production by 2.3- and 2.1-fold as compared to their parent compound BA. Other BA derivatives had comparable NO inhibition effects by reducing the NO production by 9.2%–25.6%. Given that BV2 cells are murine brain neuroglia cells and BA derivatives may not be able to penetrate the brain-blood barrier due to their molecular weight, we further evaluated the anti-inflammatory effects of BA derivatives using a human monocytic cell line (i.e., differentiated THP-1 cells).

### BA Derivatives Reduce the Production of Pro-Inflammatory Cytokines in LPS-Stimulated THP-1 Cells

**FIGURE 5:**
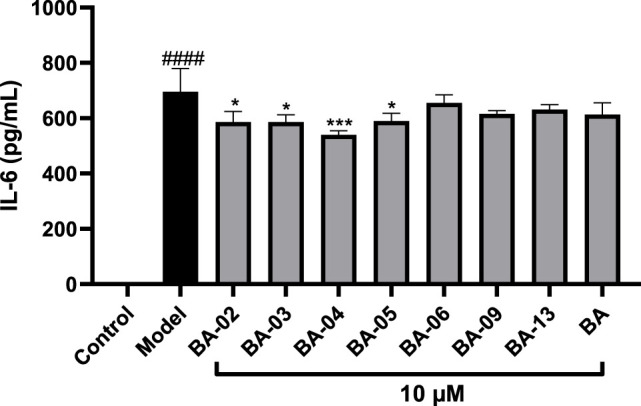
Effect of BA derivatives on the production of IL-6 in THP-1 cells. All data expressed as mean ± standard error (*n* = 3), significance was reported by analysis of variance (ANOVA) followed with Dunnett multiple comparison testing. Significance as compared to the control group *p* ≤ 0.0001 (^####^) and as compared to the model (LPS-stimulated) group, *p* ≤ 0.05 (∗) and *p* ≤ 0.001 (∗∗∗).

To further evaluate the anti-inflammatory effects of BA and its derivatives, compounds with significant inhibitory effects on NO production, namely, BA-02∼BA-06, BA-09, and BA-13, were selected for the further evaluation of their inhibitory effects on the production of pro-inflammatory cytokine IL-6 in LPS-stimulated human monocyte THP-1 cells. As shown in Figure 5, IL-6 levels in THP-1 cells were elevated to 695.6 pg/mL after stimulation with LPS. Treatment with BA and its derivatives (BA-02, BA-03, BA-04, and BA-05) at a concentration of 10 μM significantly decreased the LPS-induced secretion of IL-6 to 613.3, 586.6, 586.0, 539.6, 589.8 pg/mL, respectively.

### BA Derivatives Decreased NO Production in HA Fragment-Stimulated THP-1 Cells

**FIGURE 6:**
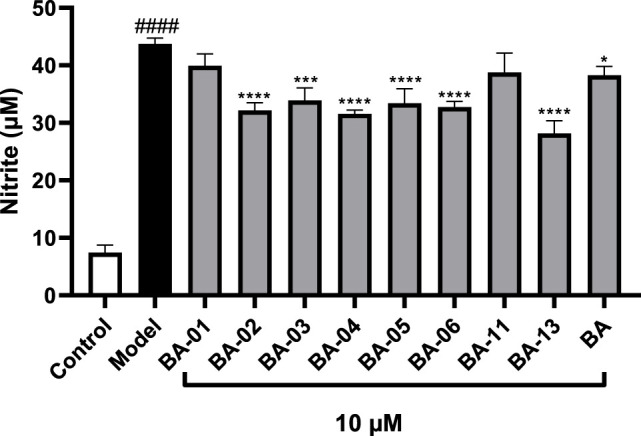
Effect of BA derivatives on the production of IL-6 in hyaluronic acid fragment stimulated THP-1 cells. All data expressed as mean ± standard error (*n* = 3), significance was reported by analysis of variance (ANOVA) followed with Dunnett multiple comparison testing. Significance as compared to the control group *p* ≤ 0.0001 (^####^) and as compared with the model group, *p* ≤ 0.05 (∗), *p* ≤ 0.001 (∗∗∗) and *p* ≤ 0.0001 (∗∗∗∗).

Given that low molecular weight HA fragments (LMWHAFs) generated from the degradation of HA are pro-inflammatory molecules (Litwiniuk et al., 2016), it is possible that BA based HAase inhibitors may exert anti-inflammatory effects by mitigating LMWHAFs-induced inflammation. Based on the data from the anti-inflammatory assays, BA and several of its derivatives including BA-01∼BA-06, BA-11, and BA-13, were selected for further anti-inflammatory effects against LMWHAFs-induced inflammation in THP-1 cells. As shown in Figure 6, the NO level of cells stimulated with LMWHAFs was elevated as compared to the control group (43.7 vs. 7.4 μM, respectively). The pro-inflammatory effect of LMWHAFs was counteracted by the treatment with BA and its derivatives BA-01∼BA-06, BA-11, and BA-13 (at a concentration of 10 μM) by reducing the LMWHAF-stimulated NO production by 12.4%, 8.6%–27.8%, 11.3% and 35.6%, respectively). The molecular mechanisms of BA derivatives’ effects against LMWHAFs-induced inflammation are still not clear but several cellular signaling pathways may be involved in the mechanisms of BA derivatives’ action. For instance, LMWHAFs can be recognized by toll-like receptors (TLRs), which are a group of protein molecules in the immune system with functions of detecting bacteria and viruses and initiating early host defense against these pathogens (Scheibner et al., 2006). It has been reported that LMWHAFs can bind to the TLR-2 and -4 receptors and consequently mediate a series of signaling events, which lead to various inflammatory responses including the generation of pro-inflammatory cytokines and chemokines in immune cells (Scheibner et al., 2006; Litwiniuk et al., 2016). Notably, in a study using an animal model of collagen induced arthritis (using female albino rats), BA was reported to block the expression of TLR-2 and -4, and reduce pro-inflammatory markers including IL-1β, tumor necrosis factor-α, and interferon-γ (Mathew and Rajagopal, 2017). It is possible that BA and its derivatives can inhibit LMWHAFs-induced inflammation in THP-1 cells via the regulation of TLRs related signaling pathways but further studies are warranted to confirm this.

## Conclusion

In summary, a series of novel BA derivatives containing an α,β-unsaturated ketene moiety were synthesized and their anti-HAase and anti-inflammatory activities were evaluated. BA derivatives with a carboxylic acid group located at the C-28 position of the BA skeleton showed enhanced inhibitory effects on HAase activity as compared to their parent compound. The preliminary SAR observation was supported by data from molecular docking assays. BA based HAase inhibitors also showed promising anti-inflammatory effects in assays using multiple cell lines. Furthermore, BA derivatives’ anti-inflammatory effects against HA fragment induced inflammation were studied in a cell-based assay. Findings from the current study supported that the chemical modifications of BA yielded novel derivatives with enhanced HAase inhibitory and promising anti-inflammatory effects but further studies are warranted to elucidate their mechanism of action.

## Data Availability

Publicly available datasets were analyzed in this study. This data can be found here: https://www.rcsb.org/, 2PE4.
